# A Georgia Doctor in New York

**Published:** 1887-01

**Authors:** K. P. Moore

**Affiliations:** New York


### A GEORGIA DOCTOR IN NEW YORK.

COMMON SENSE IN MEDICINE—ANTISEPTICS—TREATMENT OF BOWLEGS—SALICYLATES IN THE TREATMENT OF RHEUMATISM, ETC.

*Editors Atlanta Medical and Surgical Journal:*

It will be remembered that in my inaugural address before the Medical Association of Georgia, several years ago, I used as a title for my address, "Common Sense *vs.* Æstheticism, so-called." The idea which I endeavored to emphasize all through that address was the exercise of *common sense and judgment* in every department of professional life.

It is pleasing and gratifying to me to be able to note the fact that the rapid and wonderful advancement made in all directions in our profession for the last several years has been in the direction of simplicity and common sense. So-called scientific knowledge has for a long time been the veil which has covered a multitude of ignorance, and the blind faith with which we have always followed our leaders has been the price paid for thousands of lives. To illustrate: Listerism, in its broadest sense, all will admit, has saved, and is now saving thousands of lives, and yet, after all, it is still debatable as to whether it is not the soap and brush instead of the antiseptic lotions which has so revolutionized modern surgery, the exercise of common sense, in simple and simon pure cleanliness. There can be no harm in the so-called germicides, and we would be almost criminal to neglect them, but the same scrupulous use of pure water, clean towels, clean hands, clean instruments, clean sponges, clean ligatures, and constant irrigation, might accomplish the same thing. Only a few days ago I heard one of the coming surgeons of New York, and an enthusiastic advocate of antisepsis, say that just in proportion to the amount of cleanliness exercised in an operation, just in that same proportion could the strength of the antiseptic solution be reduced. This man keeps his operations irrigated all the time, and washes his hands constantly in a solution of bichloride mercury, never allowing blood to accumulate or dry on his hands. I did not intend to allow myself to be led off on to antiseptics, but before quitting this subject, I will say that every one here has become so extremely cautious that not even a silver probe is allowed to go into a wound or sinus without first having been dipped into some antiseptic; and one would be held criminal to pass the sound into the uterus, or finger into the vagina, without antiseptic precautions. As further illustrating my position, that as the profession advances step by step to a higher and more useful plane, it is only approaching a plane of simplicity, and applying the rules of common sense, we have but to recur to the plan now pursued for the cure of natural deformities of long bones. Formerly the plan for the cure of bow-legs, for instance, was the long and cruel effort of torturing the bones into shape by means of splints and screws. Then, only a few months back, osteotomy was practiced, the bone was cut down upon, a V shaped piece of bone was chiseled out, the bone was then broken and put into shape. This was approaching the principles of simplicity, but why make a compound fraction? What is the sense of complicating the fracture with an external wound? At Rosevelt, only a few days ago, I saw Dr. Sands apply the osteoclast and break several legs, and the same thing was accomplished as if osteotomy had been done; there was no injury done to the soft parts, no loss of blood, and as a natural result, less complication and speedier recovery. There was no loss of contiguity, and while patient was still under the anæsthetic, and muscles relaxed, plaster dressing was applied, and the next day patient sat up in bed, played with toys as happy as a lark and goes on to easy and quick recovery. Again, the most simple application of common sense is in the little instrument known as Banks's filiform bougie. We all can recall times when we have worried for days trying to dilate an old, inveterate stricture, when we have taxed our ingenuity and the patient's patience trying all the means at our command, and making but little satisfactory progress.

I well recall one case in which it took me three days to get down three ordinary filiform bougies, putting in one and leaving it fifteen or twenty hours, and then passing another down beside it, and so on till I could begin a small steel sound, and thus I was three weeks accomplishing what I could now accomplish with Banks's filiform in thirty minutes. Banks's filiforms and cocaine now are masters of the situation in most strictures. The only surprising thing about these filiforms is that we didn't think of the same thing a hundred years ago. By the way, I am proud of Banks because he came from Georgia.

Apropos of the discussion of cocaine among the Georgia eye doctors, it might not be uninteresting to your readers to report a discussion on the constitutional effects and uses of the drug before the Neurological Society of this city a few days ago. I enjoyed the ventilation of this question, and especially as it was handled by a man of so high standing as Dr. Hammond, of this city, who was the essayist for the occasion. He reported at length the result of some careful and searching experiments with this drug, a good many of which were made upon himself. At intervals of one or two nights he took one, two, four, eight, eighteen grains of muriate of cocaine hypodermically. He describes its effects as exceedingly pleasant and exhilarating, indeed, intoxicating, when as much as eight grains were taken at one injection, and that eighteen grains caused a temporary loss of consciousness and aberration. It produced marked acceleration and some depression of the heart's action. Its effects are evanescent—passing off in from a half hour to twelve or fifteen hours, according to amount taken. Therapeutically, he regards it as not only a pleasant and mild exhilarant, but as a fine nerve tonic and digestivant, and that it is destined to fill an important place in the materia medica. He urges caution in the preparations to be used, and recommends as a tonic and digestivant a wine of coca, prepared, according to his direction, by Thurber & Co., of this city. He regards cocaine as almost an absolutely safe remedy in ordinarily cautious hands, and does not believe that the long continuous use of it will beget the habit of using it. It does not create an irresistible desire like opium, and therefore the risk of contracting a cocaine habit is extremely small. He does not believe in the cock-and-bull newspaper stories about the socalled cocaine mania, but thinks that ninety-nine per cent. of these cases are morphine-cocaine manias. He sees how it would not only be possible, but probable, that where one had been in the habit of taking large quantities of morphia, and should undertake to substitue cocaine, the quantity that would be required to appease the opium cravings being so large, would go crazy; but he questions whether any one ever went into the cocaine habit and the concomitant cocaine mania without having first been guilty of other unpleasant and unfortunate habits.

So many valuable and interesting suggestions are caught up almost daily by one seeking them that one finds it no easy task to circumscribe a letter like this and to keep it within due bounds. But before I close I will mention one other of these, to me, valuable suggestions, viz., on the manner of giving the salicylates in the treatment of rheumatism. The same idea was gathered from two prominent teachers of different schools and on different occasions, and while it may not be a new one to many of your readers, yet to others, as to myself, it may prove worthy the space in your journal. They both accorded to salicylic acid, when combined with an alkali, decided virtues in the treatment of acute rheumatism, and attributed the failure so often met with in its use to improper administration. They recommend that it be given in doses of from 20 to 30 grs. every hour until five or six doses have been taken, or until there is great roaring or buzzing in the ears and profuse sweating. It may produce considerable prostration, and, administered in this way, will bear watching, but frequently in twenty-four to forty-eight hours the rheumatism has been abated. When given as above, after taking five or six doses hourly, then rest for six to ten hours and give four or five more doses, and so on, decreasing the number of doses during the first twenty-four to forty-eight hours.

Two months of busy, active work and observation have rapidly gone by since I came to this wide-awake metropolis. A mind thirsting for information in our dear old profession could linger for months at this fountain and not feel satiated. It is rich in all its appointments, and a real feast awaits every one who will properly utilize it. Fraternally yours, K. P. MOORE.

New York, December, 1886.

